# Supplementary material for: Increased Enzyme Loading in PICsomes via Controlling Membrane Permeability Improves Enzyme Prodrug Cancer Therapy Outcome
Source: Polymers (Basel). 2023 Mar 9;15(6):1368. doi: 10.3390/polym15061368 (PMC10057300; doi:10.3390/polym15061368)
Supplement: Supplementary file 1 [file polymers-15-01368-s001.zip › polymers-2227066-supplementary.pdf]

polymers

MDPI

Supplementary

Increased Enzyme Loading in PICsomes via Controlling Membrane Permeability Improves Enzyme Prodrug Cancer Therapy Outcome

Akinori Goto [1], Yasutaka Anraku [2,3], Shigeto Fukushima [3] and Akihiro Kishimura [4,5,*]

[1] Kyorin Pharmaceutical Co., Ltd., Watarase Research Center, 1848, Nogi, Nogi-machi, Shimotsuga-gun, Tochigi 329-0114, Japan
[2] Department of Bioengineering, Graduate School of Engineering, University of Tokyo, Hongo, Bunkyo-ku, Tokyo 113-8656, Japan
[3] Innovation Center of NanoMedicne, Kawasaki Institute of Industrial Promotion, 3-25-14 Tonomachi, Kawasaki-ku, Kawasaki 210-0821, Japan
[4] Department of Applied Chemistry, Faculty of Engineering, Kyushu University, 744 Moto-oka, Nishi-ku, Fukuoka 819-0395, Japan
[5] Center for Molecular Systems, Kyushu University, 744 Moto-oka, Nishi-ku, Fukuoka 819-0395, Japan
* Correspondence: kishimura.akihiro.776@m.kyushu-u.ac.jp; Tel.: +81-92-802-2851

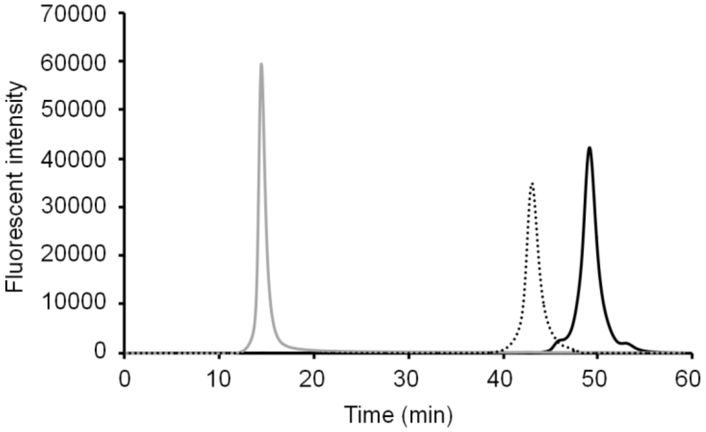

Figure S1. GPC chromatograms of Cy5-CD (dotted line), crosslinked Cy5-PICsome (gray line) and Cy5 (solid line).

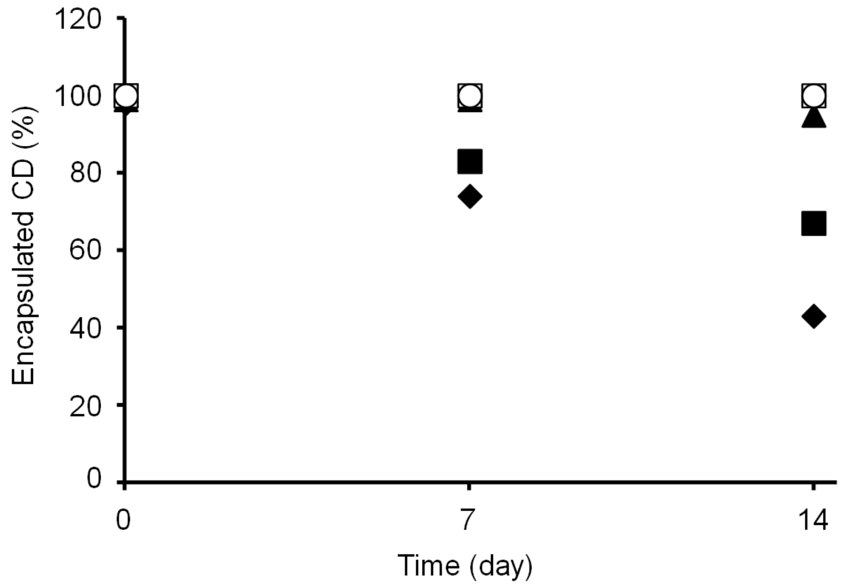

Figure S2. Cy5-CD leaking from the PICsome after the 2nd crosslinking. Second crosslinking was performed using a different concentration of EDC solutions (○: 50 mg/mL, □: 9.0 mg/mL, ▲: 5.0 mg/mL, ■: 3.0 mg/mL, and ◆: 1.0 mg/mL).

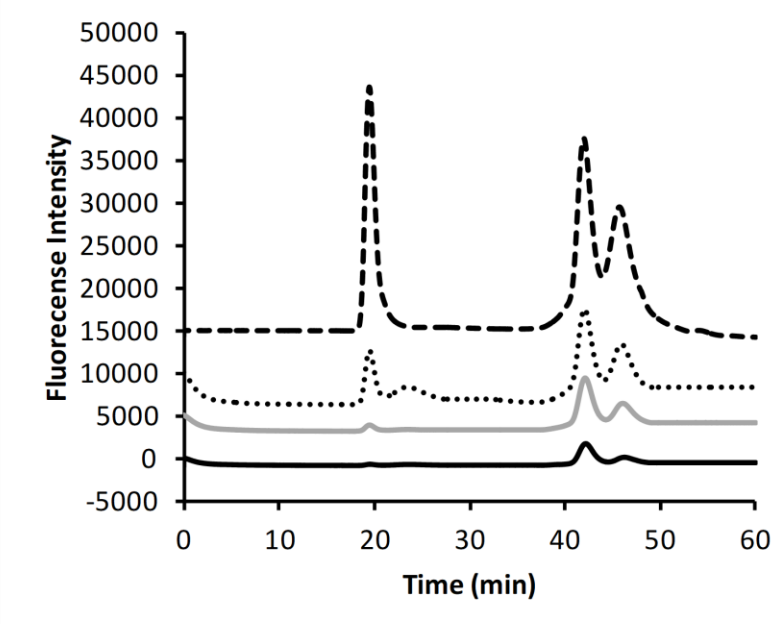

Figure S3. GPC chromatograms of the reaction mixture after the vortex-driven loading and 2nd crosslinking treatment obtained at various concentrations of Cy5-CD: 10 mg/mL (black dotted line), 5.0 mg/mL (gray dotted line), 2.5 mg/mL (gray solid line), and 1.0 mg/mL (black solid line).

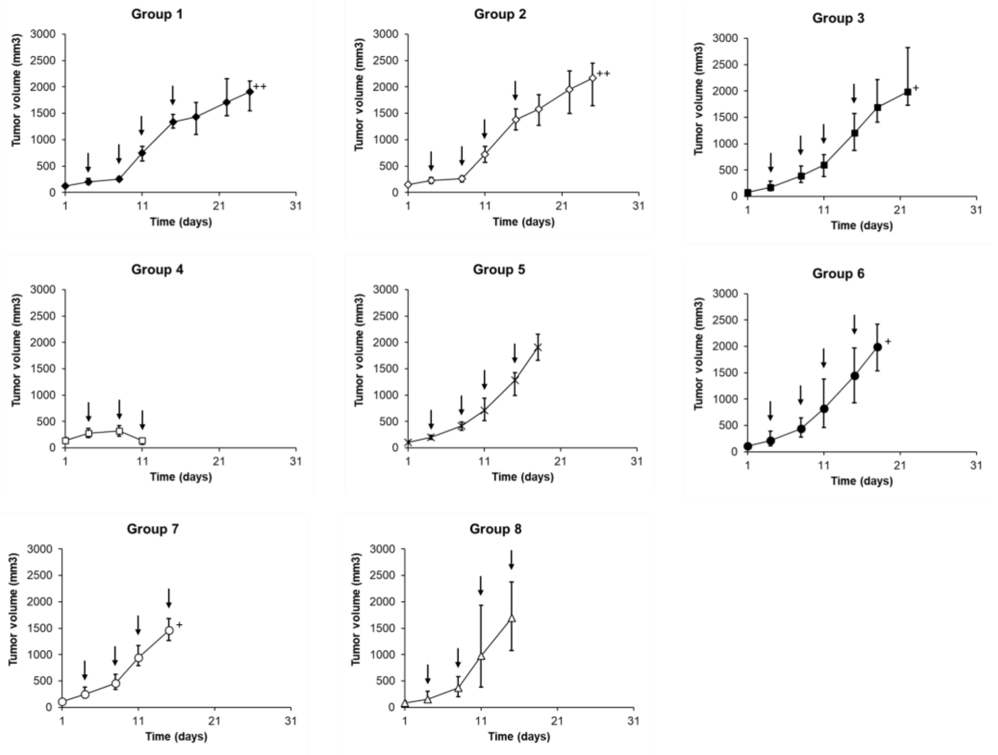

Figure S4. The tumor volume change in C26-bearing mice (n=5) treated with CD@PICsome and 10 mg/kg of 5-FC (◆, group 1), CD@PICsome and 80 mg/kg of 5-FC (◊, group 2), 10 mg/kg of 5-FU (■, group 3), 80 mg/kg of 5-FU (□, group 4) CD@PICsome alone (×, group 5), 10 mg/kg of 5-FC (●, group 6), 80 mg/kg of 5-FC (○, group 7) and 10 mM PBS (pH 6.8) (∆, group 8). An arrow indicates administration day of 5-FU or 5-FC or 10 mM PBS (pH 6.8). ++: n=3, +: n=4.

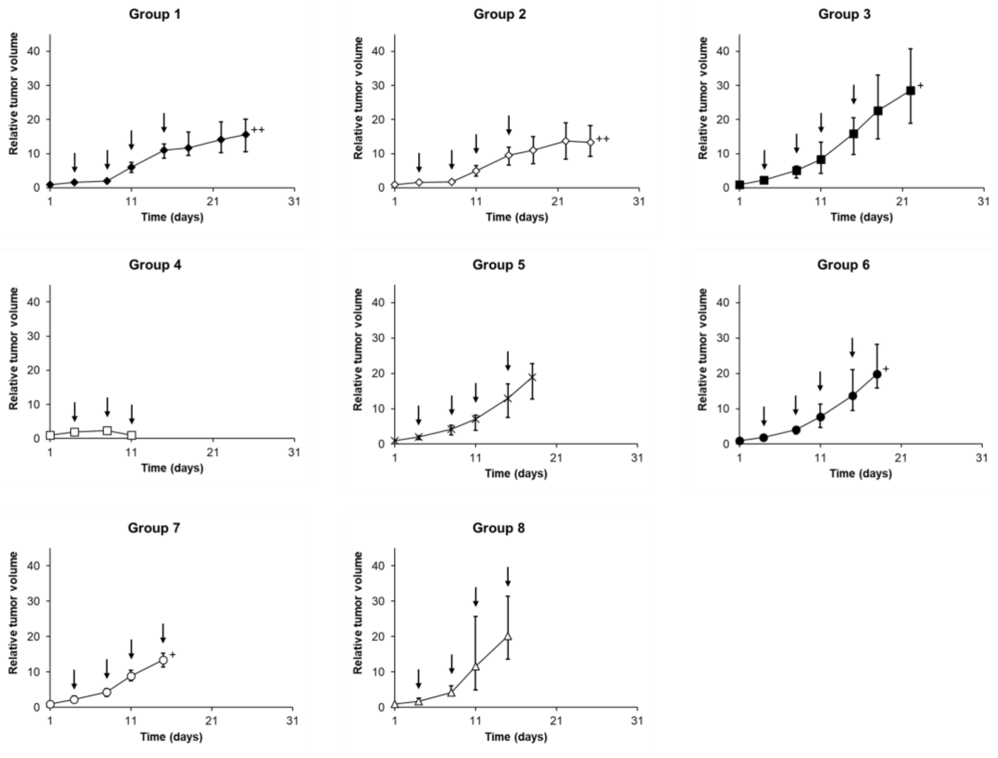

Figure S5. The relative tumor volume change in C26-bearing mice (n=5) treated with CD@PICsome and 10 mg/kg of 5-FC (◆, group 1), CD@PICsome and 80 mg/kg of 5-FC (◊, group 2), 10 mg/kg of 5-FU (■, group 3), 80 mg/kg of 5-FU (□, group 4) CD@PICsome alone (×, group 5), 10 mg/kg of 5-FC (●, group 6), 80 mg/kg of 5-FC (○, group 7) and 10 mM PBS (pH 6.8) (Δ, group 8). An arrow indicates administration day of 5-FU or 5-FC or 10 mM PBS (pH 6.8). ++: n=3, +: n=4.

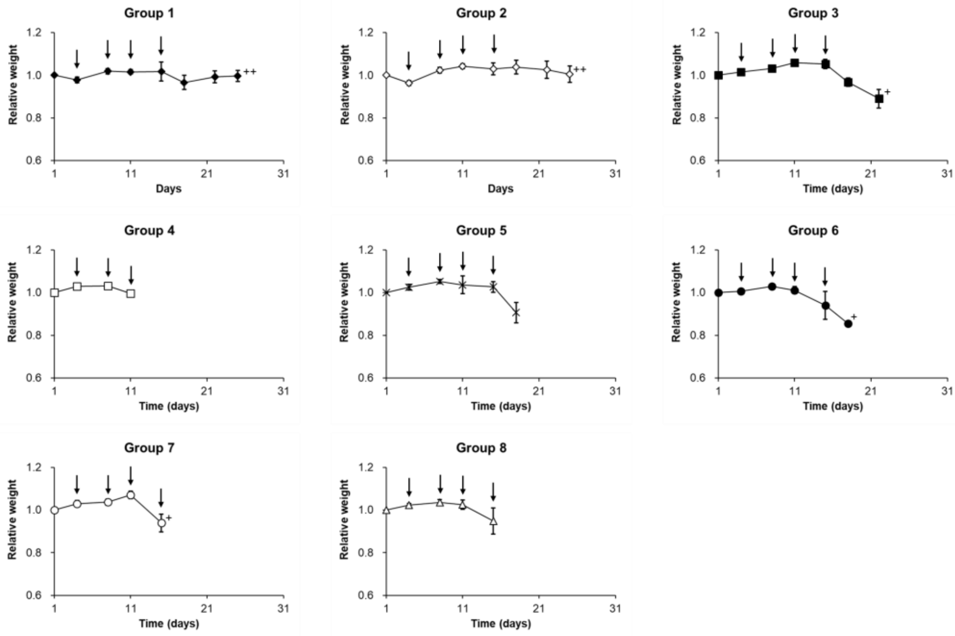

Figure S6. The weight change of mice (n=5) treated with CD@PICsome and 10 mg/kg of 5-FC (◆, group 1), CD@PICsome and 80 mg/kg of 5-FC (◊, group 2), 10 mg/kg of 5-FU (■, group 3), 80 mg/kg of 5-FU (□, group 4) CD@PICsome alone (×, group 5), 10 mg/kg of 5-FC (●, group 6), 80 mg/kg of 5-FC (○, group 7) and 10 mM PBS (pH 6.8) (Δ, group 8). An arrow indicates administration day of 5-FU or 5-FC or 10 mM PBS (pH 6.8). ++: n=3, +: n=4.
